# Renin–angiotensin–aldosterone system gene polymorphisms in gestational hypertension and preeclampsia: A case–control gene-association study

**DOI:** 10.1038/srep38030

**Published:** 2016-12-02

**Authors:** Xun Li, Hongzhuan Tan, Shujin Zhou, Shimin Hu, Tianyi Zhang, Yangfen Li, Qianru Dou, Zhiwei Lai, Fenglei Chen

**Affiliations:** 1 Xiangya School of Public Health, Central South University, 90 Xiangya Road, Changsha, Hunan, China; 2 Liuyang Municipal Hospital of Maternal and Child Health, 53 Beizheng North Road, Liuyang, Hunan, China

## Abstract

Pregnancy-induced hypertension (PIH, including preeclampsia [PE] and gestational hypertension [GH]) and cardiovascular diseases (CVDs) have some metabolic changes and risk factors in common. Many studies have reported associations between single nucleotide polymorphisms (SNPs) of renin–angiotensin–aldosterone system (RAAS) genes and CVDs (particularly hypertension), and their findings have provided candidate SNPs for research on genetic correlates of PIH. We explored the association between hypertension-related RAAS SNPs and PIH in a Chinese population. A total of 130 cases with PE, 67 cases with GH, and 316 controls were recruited. Six candidate SNPs of the RAAS system were selected. Multiple logistic regression analysis adjusting for maternal age, fetal sex, and gestational diabetes mellitus showed significant associations between angiotensinogen (AGT) rs3789678 T/C and GH (p = 0.0088) and between angiotensin II receptor type 1 (AGTR1) rs275645 G/A and PE (p = 0.0082). The study population was further stratified by maternal age (<30 and ≥30 years), and stratified and crossover analyses were conducted to determine genetic associations in different age groups. Our findings suggest that the impacts of different SNPs might be affected by maternal age; however, the effect of this potential gene–age interaction on PIH needs further exploration.

Pregnancy-induced hypertension (PIH, including preeclampsia [PE] and gestational hypertension [GH]) and cardiovascular diseases (CVDs) share some risk factors and metabolic correlates such as obesity, elevated blood pressure, insulin resistance, hyperglycemia, endothelial dysfunction, hyperlithuria, inflammation, and thrombosis^1^,^2^,^3^. In addition, women with a history of PE are more susceptible to chronic hypertension and CVDs^4^,^5^. Such similarities between PIH and CVDs suggest that they may share common mechanisms^1^. The typical manifestation of GH and PE is blood pressure elevation. Because the renin–angiotensin–aldosterone system (RAAS) is pivotal in regulating blood pressure and volume, dysfunction of this system may be one of the major underlying causes of PIH^6^. In fact, many studies have explored the association between single nucleotide polymorphisms (SNPs) of RAAS-related genes and CVDs (particularly hypertension), providing candidate SNPs for research on the genetic correlates of PIH.

Ji *et al*.^7^ studied 41 tagSNPs in RAAS and found that the occurrence of hypertension among the Chinese Han population was associated with angiotensinogen (*AGT*) rs3789678 and rs2493132, angiotensin converting enzyme (*ACE*) rs4305, and angiotensin II receptor type 1 (*AGTR1*) rs275645. However, to date, no association between the abovementioned SNPs and PIH has been reported in this population. Although *AGT* rs699 and *AGTR1* rs5186 have been extensively studied for their association with PIH and a meta-analysis^8^ showed that they are significantly associated with PE, the high heterogeneity among studies necessitates additional confirmation.

GH and PE are both characterized by *de novo* hypertension after 20 weeks of gestation; PE is hypertension with new-onset proteinuria, and GH is hypertension without proteinuria^9^. It is widely accepted that GH and PE have a shared mechanism, but it remains unknown whether they are separate diseases with similar presentations or different types of the same disorder^10^. Furthermore, little is known about why some patients with *de novo* hypertension progress to PE while others do not^11^. Therefore, investigating the genetic risk factors for GH and PE in the same population might provide a better understanding of their etiologic mechanisms.

We investigated the association of PIH with six RAAS SNPs, namely *ACE* (17q23.3) rs4305 A/G, *AGT* (1q42.2) rs2004776 G/A, rs3789678 T/C, rs699 T/C, *AGTR1* (3q24) rs275645 G/A, and rs5186 C/A.

## Results

### Demographic and clinical characteristics

A total of 197 cases, including 67 with GH and 130 with PE, as well as 316 controls were analyzed. The clinical characteristics of cases and controls are summarized in Table 1. For between-group comparisons, the Bonferroni correction was used, and α was set at 0.0167. As shown in Table 1, the maternal age and in-hospital blood pressure of women with GH and PE were significantly higher than those in normal women (p < 0.0001). The gestational age and newborn weight in the PE group were significantly lower than those in the control group (p < 0.0001 and p = 0.0164, respectively), whereas there were no significant differences between the GH and control groups. The incidence of gestational diabetes mellitus (GDM) was 11.94% in the GH group and 9.52% in the PE group, which were significantly higher than that in the control group (3.16%, p = 0.0059 and p = 0.0072, respectively). Comparisons between the GH and PE groups showed that the newborn weight in the PE group was significantly lower than that in the GH group (p = 0.015). No other differences were observed between groups.

### SNPs and PIH

The SNP detection rate was 99%. For all of the SNPs, Hardy–Weinberg equilibrium was observed in both the case and control groups. Table 2 shows the distribution of alleles among the three groups. The results of the Chi-square test showed that the distribution of *AGTR1* rs275645 G/A was significantly different in the PE and control groups (p = 0.021, α was set at 0.05 to identify potential correlations), but no significant difference in the *AGTR1* rs275645 genotype was observed between the GH and control groups.

The simple logistic regression results are shown in Table 3. No significant associations were observed between the tested SNPs and the GH/PE groups (p > 0.01). However, after adjusting for maternal age, fetal sex, and GDM (Table 4), two SNPs were significantly associated with GH or PE. Specifically, *AGT* rs3789678 was significantly associated with GH (TT vs. TC, p = 0.0088, odds ratio [OR] = 6.331, 99% confidence interval [CI]: 1.031, 38.862), and *AGTR1* rs275645 was significantly associated with PE (GG vs. GA, p = 0.0082, OR = 0.174, 99% CI: 0.032, 0.957) (Table 4).

In multiple logistic regression analysis (adjusting for maternal age, fetal sex, and GDM) of *AGT* rs3789678 and *AGTR1* rs275645, only maternal age was significantly associated with GH (p < 0.0001, OR = 1.324, 95% CI: 1.165, 1.504) or PE (p < 0.0001, OR = 1.198, 95% CI: 1.101, 1.304). To explore the potential associations of SNPs with GH/PE in different age groups, maternal age was stratified into two groups (<30 or ≥30 years), and crossover analysis was conducted for *AGT* rs3789678 and *AGTR1* rs275645. Table 5 shows the results for *AGT* rs3789678 and GH. In the <30-years age group, the *AGT* rs3789678 TT genotype was positively associated with GH (TT vs. TC, p = 0.0273, OR = 4.800, 95% CI: 1.193, 19.319). In the ≥30-years age group, the OR for the TT genotype could not be estimated due to the small number of cases. The results of the crossover analysis showed that, compared with the <30-year-old TC genotype group (reference group, OR = 1), the <30-year-old TT group and the ≥30-year TC/CC group were positively associated with GH (p < 0.05). The ≥30-year-old CC genotype group had the highest risk of GH (p < 0.0001, OR = 9.927, 95% CI: 3.521, 27.992). The results of the stratified and crossover analyses of *AGTR1* rs275645 and PE are presented in Table 6. In the <30-year-old age group, the *AGTR1* rs275645 GG genotype was negatively associated with PE (GG vs. GA, p = 0.0487, OR = 0.288, 95% CI: 0.081, 0.993); however, this association was not observed in the ≥30-year-old group. The results of the crossover analysis showed that, compared to the <30-year-old GA genotype group, those in the <30-year-old GG genotype were less likely to exhibit PE (GG vs. GA, p = 0.0487, OR = 0.288, 95% CI: 0.081, 0.993), and ≥30-year-old patients with GA or AA genotypes were more likely to show PE (p < 0.05, OR > 1). Thus, the effects of age on PE risk were not observed in the ≥30-year GG group. For *AGTR1* rs275645, those with the AA genotype who were ≥30 years of age had the highest risk for PE (p = 0.0002, OR = 6.066, 95% CI: 2.346, 15.683), and those <30 years of age in the GG genotype had the lowest risk (p = 0.0487, OR = 0.288, 95% CI: 0.081, 0.993).

For the linkage disequilibrium analysis, pairwise R^2^ values for *AGT* rs2004776, rs3789678, and rs699 ranged from 0.159 to 0.262, and R^2^ values for *AGTR1* rs275645 and rs5186 ranged from 0.189 to 0.216, suggesting low linkage disequilibrium. Haplotype analyses showed no significant associations.

## Discussion

Although the pathogenesis of PE is still unclear, many studies have suggested involvement of the RAAS^12^. The compensatory mechanism of this system is pivotal for regulating water and salt balance and for sufficient placental perfusion^6^. In a normal pregnancy, the capacity of the maternal plasma dramatically increases, and the serum levels of nearly all RAAS components increases^6^. However, these levels are significantly different in women with PE, because the RAAS balance in the circulatory system and placenta is disturbed, and maternal vascular resistance increases^6^. However, the specific mechanism underlying the pathogenesis of this condition requires additional research.

The major RAAS components include renin, angiotensinogen (AGT), angiotensin-converting enzyme (ACE), angiotensin I (AngI), AngII, angiotensin II receptor type 1 (AT1R), and AT2R. Renin catalyzes the AGT lysis to AngI, and ACE converts AngI into bioactive AngII. AngII is an octapeptide, and through its interaction with AT1R or AT2R, it is involved in vasoconstriction, sympathetic activity, cell viability, and aldosterone release^6^. The RAAS regulates blood pressure and the water–electrolyte balance through both intravascular and endocrine pathways^13^. Evidence addressing the role of RAAS genetic variants in the pathological process of CVDs was reviewed by Gluba *et al*.^14^. SNPs of RAAS members are candidates for gene-association studies regarding hypertension and PIH.

The association between *AGT* rs699 and PIH has been extensively studied. A meta-analysis^8^ that included 27 studies showed that *AGT* rs699 was significantly associated with PE (OR = 1.26, 99% CI: 1.00, 1.59), but the included studies had high heterogeneity (I^2^ = 70%). Another meta-analysis^15^ investigated the association between *AGT* rs699 and PIH in Chinese population and found significant associations in dominant genetic model, recessive genetic model, and allelic model. Although it is widely accepted that GH and PE should be analyzed separately for their different genetic backgrounds^16^, most gene-association studies performed in China have not differentiated between the two diseases; therefore, meta-analyses could not separately pool the results. In our study, no significant association was observed between *AGT* rs699 and GH or PE.

Ji *et al*.^7^ reported that the *AGT* rs3789678 polymorphism was associated with hypertension in the Han Chinese population, but the effects of different genotypes were not reported. We found that the TT (vs. TC) genotype at *AGT* rs3789678 might increase the risk for GH (adjusted OR [adOR] = 6.331, p = 0.0088, 99% CI: 1.031, 38.862), but this association was not significant (p > 0.01) before adjusting for maternal age, fetal sex, and GDM. Multiple logistic regression analysis showed that maternal age was significantly associated with GH (p < 0.001); therefore, we stratified the study population into two groups by maternal age (<30 and ≥30 years) and conducted stratified analyses. In the <30-years age group, the *AGT* rs3789678 TT genotype was positively associated with GH (TT vs. TC, p = 0.0273, OR = 4.800, 95% CI: 1.193, 19.319), but in the ≥30-years age group, the OR for the TT genotype could not be estimated due to the small number of cases. Therefore, we could not determine whether the association between *AGT* rs3789678 and GH was age dependent. Bioinformatic analyses have suggested that the AGT rs3789678 polymorphism enhances the process of mRNA splicing through the creation of a new exonic splicing enhancer and destruction of the exonic splicing silencer site^17^.

Johnson *et al*.^18^ showed that the gene polymorphism at *AGT* rs2004776 is significantly associated with hypertension. Our study found no significant association between *AGT* rs2004776 and GH/PE (p > 0.01).

*ACE* rs4646994 (*ACE* I/D) is another widely studied SNP for its association with PIH^15^,^19^,^20^. A meta-analysis by Zhu *et al*.^15^ found that *ACE* I/D was significantly associated with PIH in Chinese populations, but no significant association was found between *ACE* I/D and PIH in the subgroup with large number of cases (>100 cases). Other SNPs in the *ACE* gene have been less frequently considered in terms of their associations with PIH. A study that involved 86,588 subjects reported a significant association between *ACE* rs4305 and the risk for hypertension (p = 3.0 × 10^−5^), which is in accordance with Ji *et al*.^7^, who also reported this association in a Chinese Han population. Therefore, we investigated the association between *ACE* rs4305 and PIH, but no significant association was observed.

Ji *et al*.^7^ also reported a significant association between *AGTR1* rs275645 and hypertension; similarly, in our study, we found that the *AGTR1* rs275645 GG genotype may reduce the risk for PE compared to the GA genotype (p = 0.0082, adOR = 0.174, 99% CI: 0.032, 0.957). The results of crossover analyses further showed that the risk effects of age (≥30 years) were not significant in the GG genotype group, and women <30 years old with the GG genotype had the lowest risk for PE (p = 0.0487, OR = 0.288, 95% CI: 0.081, 0.993).

The association between *AGTR1* rs5186 and PE has been studied extensively, but the results have been inconsistent^21^,^22^,^23^. A meta-analysis^8^ covering 10 studies found no significant association (OR = 1.22, 99% CI: 0.96–1.56); similarly, our study found no association of *AGTR1* rs5186 with PE or GH.

Age is an important predictor of PIH and CVDs, although it remains unknown if the genetic effects are stable or change with age. Many studies have indicated that gene–age interactions may be related to the dynamic processes of gene expression and protein modification^24^. Age-related behaviors and environmental exposure may also affect epigenetic modifications, such as changes in DNA methylation status and aberrant micro-RNA expression^25^. The generation of reactive oxygen species and the oxidative damage that they create increase with age, and may affect the post-translational modification of proteins involved in gene regulation^26^. The interaction between age and genes has been reported in various studies. For example, a genome-wide association study that involved 1240 subjects showed that the expression of more than 4300 human genes was age dependent, and an interaction with age was found in at least 623 of these genes^27^. In addition, studies have reported the effects of gene–age interactions on blood pressure^28^,^29^. We conducted stratified and crossover analyses to explore the associations between SNPs and GH/PE in different age groups. Our results for *AGTR1* rs275645 were intriguing. In the <30-years age group, the *AGTR1* rs275645 GG genotype was negatively associated with PE (GG vs. GA, p = 0.0487, OR = 0.288, 95% CI: 0.081, 0.993), but this association was not observed in the ≥30-years age group. Crossover analyses showed a negative association with PE in women <30 years old who harbored the GG genotype (GG vs. GA, p = 0.0487, OR = 0.288, 95% CI: 0.081, 0.993), whereas carrying the GA or AA genotypes and being ≥30 years old increased the risk of PE (p < 0.05). However, additional studies are needed to confirm whether the age-related risk for PE is compensated by the protective effects of the GG genotype.

The current gene-association study was limited by the small sample size, which makes it less likely to identify very weak associations; this is a common problem in this type of study. Due to limitations imposed by our sample source, we could not collect the same number of GH and PE cases, which may have resulted in differences in statistical power. In addition, our GH sample size was relatively small. We found that *AGT* rs3789678 was significantly associated with GH, but not with PE, whereas *AGTR1* rs275645 was significantly associated with PE, but not with GH. However, we were unable to determine whether these differences were due to actual dissimilarities in the two diseases or differences caused by the small sample size. Data analyses from the National Hospital Discharge Survey (United States, 1979–1986) indicate that the risk of PE increases by 30% for every additional year of age past 34^30^. Because the number of pregnant women ≥35 years old was very small (27 of 197), we stratified the population by a maternal age of 30 rather than 35; however, even with this stratification, the sample size of some genotypes was very small (n < 10). Because the estimation of gene–age interactions requires large sample sizes to achieve a reasonable statistical power^31^,^32^, we did not further explore these interactions in the present study. In addition to maternal age, fetal sex, and GDM, many other factors have been identified as risk factors for PE and/or GH, such as a history of PE and infertility^33^, body mass index above 24, and primiparity^34^. We did not adjust for these factors in the multiple logistic regression because the proportion of missing data for those factors was larger than 15%. Another limitation of our study is that we did not conduct subgroup analyses according to early- or late-onset PE because the gestational age of PE onset was not regularly recorded in the medical records of our study region. We also did not categorize PE cases by severity. Of the 130 cases of PE, 36 (27.69%) were diagnosed with severe PE, but we were not able to collect corresponding clinical lab results to verify those diagnoses.

From above, our study showed that hypertension-related SNPs were associated with PE or GH in a Han Chinese population. The *AGT* and *AGTR1* genes may be involved in common elements of the pathogenesis of hypertension, PE, and GH. These results also provide genetic evidence to support that patients with PE or GH might have a higher risk for hypertension. Our data encourage further research exploring the similarities and differences in disease-related genes between GH and PE. Similar pathogenic genes will reveal the similarities between these two diseases, whereas different genes may not only provide important clues for pathogenic research but also help in the prediction of PIH. Finally, additional studies are needed to determine the effects of gene–age interactions on this disease.

## Methods

### Study population

Subjects were recruited from the Liuyang Municipal Hospital of Maternal and Child Health, Hunan Province of China. The inclusion criteria for the case group were clinical diagnosis of GH or PE combined with the absence of diabetes mellitus, renal disease, CVDs, or other diseases that are already known as risk factors for GH and PE. The controls were healthy women (without GH, PE, and the other aforementioned diseases) who delivered at the same hospital during the study period. All of the subjects provided written informed consent, after which blood samples and medical records were collected. A total of 130 patients with PE, 67 with GH, and 316 controls were recruited.

### Ethics statement

The study protocol was reviewed and approved by the Central-South University’s Ethical and Confidentiality Committee. All participants provided written informed consent. The authors assert that all of the procedures/methods were performed in accordance with the approved guidelines.

### Diagnostic criteria

Some diagnostic criteria^35^,^36^ recommend a broad definition of PE, namely, that diagnosis of PE should include *de novo* hypertension accompanied by other maternal organ dysfunction or uteroplacental dysfunction even in the absence of proteinuria. However, there is still no clear consensus on the classification of this disease. The International Society for Study of Hypertension in Pregnancy recommends a broad definition of PE in the clinic but a strict definition in scientific research because the inclusion of proteinuria ensures a more specific diagnosis^9^. Therefore, we used the strict definition, defining PE as *de novo* hypertension (systolic blood pressure ≥140 mm Hg and/or diastolic 90 mm Hg) after 20 weeks of gestation accompanied by proteinuria (urinary protein dip sticks ≥2+ or ≥300 mg in a 24-h urine sample). GH was similarly defined as *de novo* hypertension, but without the presence of proteinuria^37^.

### SNP selection and genotyping

Six candidate SNPs of the RAAS system were selected, including *ACE* (17q23.3) rs4305 A/G, *AGT* (1q42.2) rs2004776 G/A, rs3789678 T/C, rs699 T/C, *AGTR1* (3q24) rs275645 G/A, and rs5186 C/A.

Genomic DNA was extracted from whole blood using the TIANamp Blood DNA Kit (DP318-03, TIANGEN, Beijing), which is based on silica membrane technology and uses a special buffer system for DNA extraction from fresh or frozen whole blood. SNPs were genotyped with the SEQUENOM MassARRAY iPLEX platform^3^,^8^. The assay consists of an initial locus-specific PCR reaction, followed by single base extension and matrix-assisted laser desorption/ionization–time of flight mass spectrometry to identify the SNP allele^38^.

### Statistical analysis

Case–control studies were conducted to compare the PE and control groups and the GH and control groups. General clinical features of case and control groups were compared with the *t*-test or Wilcoxon rank sum test for continuous variables, and the Chi-square test was used for categorical variables. The Bonferroni correction^39^ was applied for multiple comparisons (α = 0.05/3 = 0.0167).

The SNP detection rate was calculated as the number of sites that were successfully genotyped for all of the samples divided by the number of genotyped sites for all of the samples. The Hardy–Weinberg test was conducted for the case and control groups using the Chi-square goodness-of-fit test or the Fisher’s exact test^40^ (α = 0.01). The Chi-square test was used to test the genotype distribution between the case and control groups. Because this was an exploratory study, a p-value of 0.05 was used to identify potential correlations. Logistic regression was used to estimate the OR. α was set at 0.01 to control for any type Ι errors that may occur with multiple testing, and the 99% CI was calculated for different genetic models. The OR and 99% CI were calculated after adjusting for known risk factors for PIH such as maternal age, fetal sex, and GDM^34^. If adjusting for other risk factors changed the significance of SNPs, stratified and crossover analyses were conducted, and logistic regression was used to estimate the OR and 95% CI for each group. Pair-wise linkage disequilibrium (R)^2^ was estimated using SHEsis^41^. Chi-square tests were used to determine whether haplotype frequency distributions differed between the case and control groups. All of the statistical analyses, except for linkage disequilibrium analysis, were performed using SAS 9.2 (SAS Institute, Inc., Cary, NC, USA).

## Additional Information

**How to cite this article**: Xun, L. *et al*. Renin–angiotensin–aldosterone system gene polymorphisms in gestational hypertension and preeclampsia: A case–control gene-association study. *Sci. Rep.* **6**, 38030; doi: 10.1038/srep38030 (2016).

**Publisher's note:** Springer Nature remains neutral with regard to jurisdictional claims in published maps and institutional affiliations.

## Figures and Tables

**Table 1:** Demographic and clinical characteristics of the study subjects.

	Controls		GH^a^			PE^a^			p*,^f^
	N = 316		N = 67		p*,^d^	N = 130		p*,^e^	
	Median^b^	(QL, QU)^c^	Median	(QL, QU)		Median	(QL, QU)		
Maternal age, years	25	(23,26)	27	(25.0,31.5)	<0.0001	26	(24.0,30.0)	<0.0001	0.1543
Gestational age at delivery (week)	39	(38,40)	39	(38,40)	0.1286	38	(37,39)	<0.0001	0.0449
New born weight (kg)	3.3	(3,3.6)	3.35	(3.10,3.75)	0.1892	3.10	(2.70,3.60)	0.0164	0.0151
In-hospital SBP^g^	110	(100,120)	140	(130,145)	<0.0001	132	(140,147)	<0.0001	0.2748
In-hospital DBP^g^	70	(60,75)	90	(85,95)	<0.0001	90	(80,95)	<0.0001	0.8955
	n/N	%	n/N	%	p**^ ^d	n/N	%	p**^ ^e	p*^ ^f
GDM^h^	10/316	3.16	8/67	11.94	0.0059	12/130	9.52	0.0072	0.9153
Fetal sex, male	165/305	54.10	26/50	52.00	0.7820	64/121	52.89	0.8219	0.5508

^*^Wilcoxon rank sum test due to a non-normal distribution of tested characteristics; α set at 0.0167 for multiple comparisons (Bonferroni correction, 0.05/3);

^**^Chi-square test or Fisher exact test. α set at 0.0167 for multiple comparisons (Bonferroni correction).

^a^PE, preeclampsia; GH, gestational hypertension.

^b^For variables that did not follow a normal distribution, median and quartiles are used for the statistical description.

^c^QL, lower quartile (25%); QU, upper quartile (75%).

^d^Comparison of GH and control group.

^e^Comparison of PE and control group.

^f^Comparison of GH and PE group.

^g^SBP and DBP: blood pressure measured after women arrived to the hospital for delivery and before entering the delivery room, respectively. PE women might have received treatment to control their blood pressure before the blood pressure measurements.

^h^GDM, gestational diabetes mellitus.

**Table 2:** Distribution of tested genotypes among cases and control subjects.

Gene	SNP	Genotype	Controls		GH			PE		
			n	%	n	%	p*	n	%	p*
*ACE*	rs4305	AA	37	11.82	9	13.64	0.534	17	13.28	0.151
		GG	110	35.14	27	40.91		56	43.75	
		AG	166	53.04	30	45.45		55	42.97	
*AGT*	rs2004776	GG	55	17.92	12	19.05	0.119	24	18.90	0.813
		AA	99	32.25	28	44.44		44	34.65	
		GA	153	49.84	23	36.51		59	46.46	
*AGT*	rs699	TT	9	2.89	1	1.52	0.400	3	2.34	0.936
		CC	215	69.13	51	77.27		90	70.31	
		TC	87	27.97	14	21.21		35	27.34	
*AGT*	rs3789678	TT	8	2.580	5	7.69	0.075	4	3.17	0.937
		CC	212	68.39	46	70.77		85	67.46	
		TC	90	29.03	14	21.54		37	29.37	
*AGTR1*	rs5186	CC	0	0	0	0	0.530	0	0	0.261
		AA	277	88.22	60	90.91		107	84.25	
		CA	37	11.78	6	9.09		20	15.75	
*AGTR1*	rs275645	GG	27	8.60	4	6.06	0.743	4	3.13	**0.021**
		AA	183	58.28	41	62.12		67	52.34	
		GA	104	33.12	21	31.82		57	44.53	

^*^Chi-square test or Fisher exact test. α set at 0.05 for exploration purposes.

**Table 3:** Simple logistic regression for RAAS SNPs.

Gene	SNP	Genotype	GH			PE		
			p	OR	99% CI	p	OR	99% CI
*ACE*	rs4305	AA vs. AG	0.4807	1.346	(0.455,3.984)	0.3244	1.387	(0.590,3.259)
		GG vs. AG	0.2951	1.358	(0.640,2.884)	0.0576	1.537	(0.858,2.751)
		AA vs. GG + AG	0.6818	1.178	(0.421,3.293)	0.6713	1.142	(0.509,2.564)
		GG vs. AA + AG	0.3763	1.278	(0.626,2.607)	0.0911	1.435	(0.827,2.490)
*AGT*	rs2004776	GG vs. GA	0.3386	1.451	(0.533,3.956)	0.6685	1.132	(0.538,2.380)
		AA vs. GA	0.0412	1.881	(0.848,4.176)	0.5497	1.153	(0.625,2.124)
		GG vs. AA + GA	0.8317	1.078	(0.434,2.681)	0.8093	1.068	(0.531,2.147)
		AA vs. GG + GA	0.0650	1.681	(0.814,3.470)	0.6288	1.114	(0.627,1.978)
*AGT*	rs699	TT vs. TC	0.7347	0.690	(0.041,11.522)	0.7871	0.829	(0.138,4.977)
		CC vs. TC	0.2359	1.474	(0.634,3.426)	0.8665	1.041	(0.566,1.912)
		TT vs. CC + TC	0.5339	0.516	(0.033,7.978)	0.7485	0.805	(0.142,4.583)
		CC vs. TT + TC	0.1900	1.518	(0.668,3.446)	0.8071	1.058	(0.586,1.908)
*AGT*	rs3789678	TT vs. TC	0.0294	4.018	(0.776,20.804)	0.7607	1.216	(0.232,6.369)
		CC vs. TC	0.3134	1.395	(0.596,3.265)	0.9147	0.975	(0.534,1.781)
		TT vs. CC + TC	0.0510	3.146	(0.693,14.282)	0.7316	1.238	(0.25,6.139)
		CC vs. TT + TC	0.7064	1.119	(0.518,2.416)	0.8502	0.958	(0.535,1.715)
*AGTR1*	rs5186	AA vs. CA	0.5316	1.336	(0.406,4.396)	0.2626	0.715	(0.33,1.547)
*AGTR1*	rs275645	GG vs. GA	0.5982	0.734	(0.162,3.327)	0.0196	0.270	(0.064,1.145)
		AA vs. GA	0.7246	1.110	(0.519,2.373)	0.0643	0.668	(0.381,1.171)
		GG vs. AA + GA	0.4963	0.686	(0.165,2.856)	0.0502	0.343	(0.084,1.401)
		AA vs. GG + GA	0.5645	1.174	(0.573,2.405)	0.2539	0.786	(0.457,1.353)

*α set at 0.01 because of multiple testing.

**Table 4:** Multiple logistic regression analysis for RAAS SNPs.

Gene	SNP	Genotype	GH			PE		
			p	adOR^a^	99% CI	p	adOR^a^	99% CI
*ACE*	rs4305	AA vs. AG	0.6851	1.249	(0.304,5.140)	0.3379	1.434	(0.544,3.774)
		GG vs. AG	0.4023	1.354	(0.533,3.443)	0.1100	1.491	(0.783,2.836)
		AA vs. GG + AG	0.8704	1.089	(0.284,4.175)	0.6133	1.198	(0.478,3.002)
		GG vs. AA + AG	0.4503	1.296	(0.535,3.143)	0.1659	1.389	(0.754,2.558)
*AGT*	rs2004776	GG vs. GA	0.1321	2.097	(0.591,7.442)	0.6663	1.149	(0.501,2.637)
		AA vs. GA	0.0373	2.295	(0.821,6.413)	0.8132	1.064	(0.540,2.097)
		GG vs. AA + GA	0.4606	1.378	(0.450,4.219)	0.7090	1.120	(0.513,2.442)
		AA vs. GG + GA	0.0941	1.805	(0.728,4.478)	0.9233	1.024	(0.542,1.936)
*AGT*	rs699	TT vs. TC	0.4525	2.377	(0.122,46.226)	0.8151	1.196	(0.167,8.591)
		CC vs. TC	0.2443	1.639	(0.550,4.886)	0.8775	0.960	(0.485,1.899)
		TT vs. CC + TC	0.6642	1.610	(0.095,27.228)	0.7786	1.232	(0.182,8.314)
		CC vs. TT + TC	0.2992	1.523	(0.536,4.327)	0.8278	0.946	(0.488,1.831)
*AGT*	rs3789678	TT vs. TC	**0.0088**	**6.331**	**(1.031,38.862)**	0.6109	0.663	(0.083,5.319)
		CC vs. TC	0.2335	1.635	(0.565,4.737)	0.5094	1.187	(0.608,2.317)
		TT vs. CC + TC	0.0182	4.449	(0.873,22.671)	0.5053	0.591	(0.078,4.509)
		CC vs. TT + TC	0.7172	1.140	(0.450,2.889)	0.4255	1.224	(0.637,2.352)
*AGTR1*	rs5186	AA vs. CA	0.3609	1.758	(0.358,8.618)	0.5081	0.799	(0.334,1.913)
*AGTR1*	rs275645	GG vs. GA	0.5994	0.712	(0.135,3.763)	**0.0082**	**0.174**	**(0.032,0.957)**
		AA vs. GA	0.9037	0.956	(0.367,2.490)	0.1471	0.703	(0.375,1.315)
		GG vs. AA + GA	0.6051	0.733	(0.156,3.441)	0.0173	0.215	(0.041,1.135)
		AA vs. GG + GA	0.9230	1.034	(0.425,2.514)	0.5820	0.879	(0.481,1.607)

*α set at 0.01 because of multiple testing.

^a^adOR, adjusted OR. ORs adjusted for maternal age, fetal sex, and gestational diabetes mellitus.

**Table 5:** Stratified and crossover analyses for *AGT* rs3789678 and GH.

Age	Genotype	Controls	GH	Stratified Analysis		Crossover Analysis	
		n (%)	n (%)	OR (95% CI)	p*	OR (95% CI)	p*
<30	TC	84 (28.00)	10 (18.18)	1 (Reference)		1 (Reference)	
	TT	7 (2.33)	4 (7.27)	4.800 (1.193,19.319)	0.0273	4.800 (1.193,19.319)	0.0273
	CC	192 (64.00)	23 (41.82)	1.006 (0.459,2.207)	0.9876	1.006 (0.459,2.207)	0.9876
≥30	TC	6 (2.00)	4 (7.27)	1 (Reference)		5.600 (1.347,23.283)	0.0178
	TT^a^	0	1 (1.82)	—	0.9775	—	0.9870
	CC	11 (3.67)	13 (23.64)	1.773 (0.396,7.932)	0.4539	9.927 (3.521,27.992)	<0.0001

^*^α set at 0.05.

^a^ORs could not be estimated by logistic regression due to the small number of cases.

**Table 6:** Stratified and crossover analyses of *AGTR1* rs275645 and PE.

Age	Genotype	Controls	PE	Stratified Analysis		Crossover Analysis	
		n (%)	n (%)	OR (95% CI)	p*	OR (95%CI)	p*
<30	GA	92 (30.26)	99 (31.45)	1 (Reference)		1 (Reference)	
	GG	25 (8.22)	3 (2.42)	0.288 (0.081,0.993)	0.0487	0.288 (0.081,0.993)	0.0487
	AA	170 (55.92)	46 (37.10)	0.638 (0.389,1.049)	0.0763	0.638 (0.389,1.049)	0.0763
≥30	GA	8 (2.63)	17 (13.71)	1 (Reference)		5.013 (1.998,12.578)	<0.0006
	GG	2 (0.66)	1 (0.81)	0.235 (0.018,2.993)	0.2648	1.179 (0.104,13.391)	0.8941
	AA	7 (2.30)	18 (14.52)	1.210 (0.360,4.065)	0.7578	6.066 (2.346,15.683)	0.0002

^*^α set at 0.05.
